# Pancreatoduodenectomy combined with portal-superior mesenteric vein resection and reconstruction with interposition grafts for cancer: a meta-analysis

**DOI:** 10.18632/oncotarget.20866

**Published:** 2017-09-14

**Authors:** Wei Song, Qifan Yang, Linghui Chen, Qiang Sun, Dongkai Zhou, Song Ye, Zhenhua Hu, Liming Wu, Liming Feng, Shusen Zheng, Weilin Wang

**Affiliations:** ^1^ Key Laboratory of Precision Diagnosis and Treatment for Hepatobiliary and Pancreatic Tumor of Zhejiang Province, The First Affiliated Hospital, College of Medicine, Zhejiang University, Hangzhou 310003, China; ^2^ Division of Hepatobiliary and Pancreatic Surgery, Department of Surgery, First Affiliated Hospital, Zhejiang University School of Medicine, Hangzhou 310003, China; ^3^ Collaborative Innovation Center for Diagnosis and Treatment of Infectious Diseases, Hangzhou 310003, China

**Keywords:** pancreaticoduodenectomy, vein resection and reconstruction, grafts, meta-analysis

## Abstract

The use of interposition grafts for portal-superior mesenteric vein (PV-SMV) reconstruction during pancreatoduodenectomy (PD) with venous resection (VR) for localized periampullary tumors is a controversial topic. The present meta-analysis aimed to evaluate the perioperative and long-term outcomes in patients who received interposition grafts for PV-SMV reconstruction after PD with VR. The correlative databases were systematically searched to identify relevant trials comparing vein grafts versus no vein grafts during PD with VR. 14 studies including 257 patients with vein grafts and 570 patients without vein grafts were extracted. The meta-analysis indicated no difference in perioperative morbidity, mortality, or thrombosis between the two groups, but the vein graft group was associated with a significantly increased venous thrombosis rate (≥ 6 months) (odds ratio [OR] = 2.75; 95% confidence interval [CI], 1.32–5.73; *P* = .007). The autologous vein group subgroup analysis also revealed a significantly increased vein thrombosis rate (OR = 3.13; 95% CI, 1.45–6.76; *P* = .004) between the two groups. Meanwhile, the prosthetic vein group subgroup analysis indicated no difference. Additionally, the oncological value of vein grafts during PD for pancreatic cancer survival was analyzed and revealed no difference in 1-year, 3-year, or 5-year survival between the two groups. Using interposition grafts for PV-SMV reconstruction is safe and effective, and has perioperative outcomes and long-term survival rates compared to those with no vein grafts during PD with VR. However, the lower long-term vein patency rate in patients with vein grafts indicate that interposition grafts may be more likely to lose function.

## INTRODUCTION

Pancreatoduodenectomy (PD) provides the only possibility for a cure or for the long-term survival of patients with pancreatic and periampullary neoplasms [1]. The close proximity of the pancreatic head to major venous structures frequently causes tumor invasion of the portal-superior mesenteric vein (PV-SMV); therefore, the major goal of surgery is radical resection and cure, which requires complete resection of the tumor with en-bloc venous infiltration [2, 3].

The poor perioperative and long-term outcomes in patients with pancreatic cancer combined with venous resection (VR) discourage surgeons from considering a more aggressive approach to increased resection rates [4–6]. Recent studies suggest that survival rates for patients undergoing PV-SMV reconstruction during PD with VR for localized periampullary tumors was comparable to those undergoing conventional PD. VR during PD is a safety of surgical procedure which has therefore no longer been considered a contraindication to resection when performed by an experienced surgeon [1, 3, 4, 7, 8].

The techniques used for PV-SMV reconstruction are classified into four main types—primary end-to-end anastomosis, venorrhaphy, patch venoplasty, and graft interposition—and remain a controversial issue [9–11]. Primary end-to-end anastomosis or direct suturing is the most common option for PV-SMV reconstruction without any interposition grafts [12–14]. Several studies have determined that PV-SMV resection can be performed with primary end-to-end anastomosis or venorrhaphy reconstruction, avoiding a vein graft [15, 16]. However, when primary end-to-end anastomosis has a risk of stenosis or over-tension of the PV-SMV reconstruction, an interposed graft is necessary [5]. Autologous veins [17, 18], prosthetic veins [11, 19], and allograft veins [20, 21] have been used in PV-SMV reconstruction, and the most appropriate interposed grafts for PV-SMV reconstruction after PD remains controversial. Because of the risk of developing a postoperative or long-term PV-SMV thrombus leading to bowel ischemia, sepsis, or death [22–24], it also remains controversial which patients are indicated for use of an interposed graft for PV-SMV reconstruction when undergoing PD with venous resection to promote postoperative and long-term PV-SMV thrombus.

A meta-analysis can extract the available evidence and help obtain more precise estimates of treatment efficacy and safety. Therefore, the aim of present meta-analysis was to estimate the perioperative outcomes and long-term survival of patients who received interposition grafts for PV-SMV reconstruction during PD with VR.

## RESULTS

### Characteristics of the studies included

**Figure 1:**
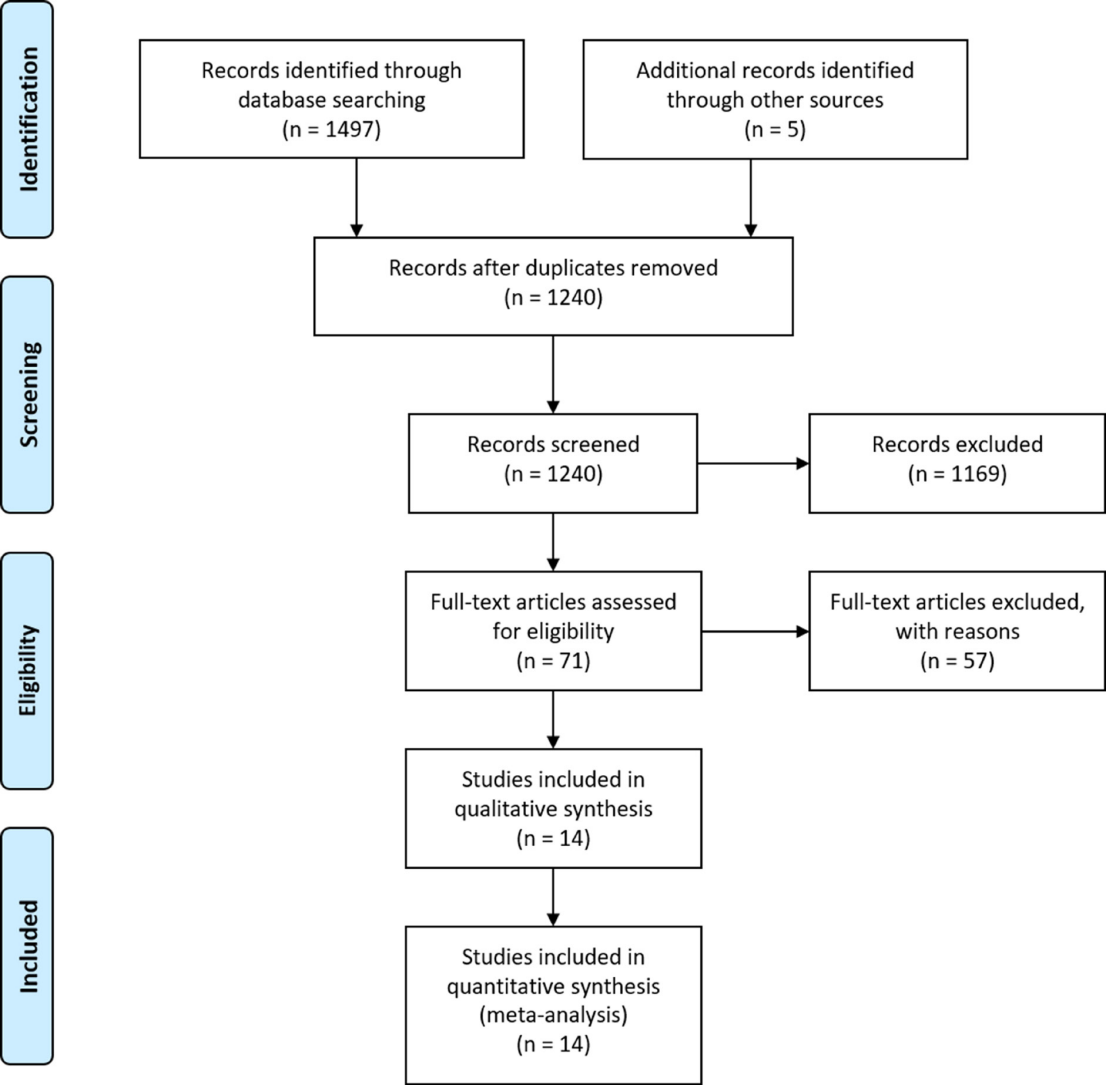
PRISMA flow diagram for the literature search

The initial literature search yielded 1240 studies. Through review of the titles and abstracts, 71 studies were identified and selected the basis of inclusion criteria, and the full texts were obtained and assessed in more detail. Of these, 51 studies were excluded due to insufficient data of key outcomes. Additionally, 5 studies lacked a control group [20, 25–29]. In 2 studies, data were extracted from the same institution [21, 28]. Finally, 14 studies were identified which matched our inclusion criteria and received an NOS score ≥ 7 and were included in the analysis [9–11, 21, 30–39] (Figure 1). The key characteristics of the included studies are shown in Table 1.

**Table 1:** Description of the 14 studies included in the meta-analysis

Study	Inclusion period	Country	Reserch type	Group	No. of patients	Length of resected (cm)	Method of reconstruction	Pathological diagnosis	NOS score
Stauffer2009 [30]	2000–2007	USA	Retrospec.	vein graft	17	n/a	AV = 7; PV = 10	PC = 27	7
				no vein graft	10	n/a	PA = 10		
Liao 2014 [11]	2007–2012	China	Retrospec.	vein graft	34	4.0 ± 0.9	PV = 34	PC = 29; AA = 1; BDC = 2; DA = 2	9
				no vein graft	42	2.9 ± 0.7	PA = 42	PC = 36; NET = 1; AA = 3; BDC = 2	
Kim 2013 [31]	2007–2012	Korea	Retrospec.	vein graft	7	n/a	AV = 2; PV = 1; Other = 4	PC = 16	7
				no vein graft	9	n/a	PA = 9		
Smoot 2006 [32]	1988–2003	USA	Retrospec.	vein graft	22	n/a	AV = 4; PV = 18	PC = 35	7
				no vein graft	13	n/a	PA = 13		
Muller 2009 [33]	2001–2007	Germany	Retrospec.	vein graft	20	n/a	AV = 4; PV = 14; PVP = 2	PC = 92	8
				no vein graft	72	n/a	PA = 72		
Kaneoka 2009 [9]	1993–2006	Japan	Retrospec.	vein graft	15	5.1 (4.0–7.0)	AV = 15	PC = 42	7
				no vein graft	27	2.6 (1.0–5.0)	PA = 27		
Hirono 2014 [10]	2000–2012	Japan	Retrospec.	vein graft	14	5.0 (3.0–7.0)	AV = 14	PC = 12; SPN = 1; SCN = 1	9
				no vein graft	114	2.0 (0.5–6.0)	PA = 103; LW = 11	PC = 107; BDC = 6; TFP = 1	
Glebova 2015 [36]	1970–2014	USA	Retrospec.	vein graft	22	n/a	AV = 11; PV = 6; PVP = 5	PC = 127	7
				no vein graft	105	n/a	PA = 105		
Wang 2015 [21]	2009–2013	China	Retrospec.	vein graft	14	4.39 (3.5–5.0)	AGV = 14	PC = 42	8
				no vein graft	28	2.56 (1.0–4.0)	PA = 28		
Dua 2015 [37]	2005–2014	USA	Retrospec.	vein graft	36	n/a	AV = 19; AVP = 17	PC = 67; NET = 17; Other = 6	7
				no vein graft	54	n/a	PA = 28; LW = 26		
Amico 2014 [38]	2007–2014	Brazil	Retrospec.	vein graft	5	4.75 ± 1.3	AVP = 3; PV = 2	PC = 10	7
				no vein graft	5	2.80 ± 1.4	PA = 5		
Leach 1998 [39]	1990–1995	USA	Retrospec.	vein graft	16	n/a	AV = 15; PV = 1	PC = 31	7
				no vein graft	15	n/a	PA = 15		
Gong 2013 [34]	2006–2011	China	Retrospec.	vein graft	43	n/a	PV = 43	PC = 94	8
				no vein graft	51	n/a	PA = 51		
Ouaissi 2008 [35]	1996–2006	France	Retrospec.	vein graft	2	4.5	PV = 2	PC = 25; BDC = 1; Other = 1	7
				no vein graft	25	1.32 (1.0–4.0)	LW = 24; PA = 1		

Abbreviations: AV, autologous vein; PV, prosthetic vein; AGV, allograft vein; PA, primary end to end anastomosis; AVP, autologous vein patch; PVP, prosthetic vein patch; PC, pancreatic cancer; NET, neuroendocrine tumor; BDC, bile duct cancer; AA, ampullary adenocarcinoma; DA, duodenal adenocarcinoma; SCN, serous cyst neoplasm; SPN, solid-pseudopapillary neoplasm; TFP, tumor-forming pancreatitis.

The present meta-analysis included 837 patients who underwent pancreatoduodenectomy with vein resection in 14 studies; of these patients, 267 patients were included into the vein graft group (autologous vein: 110, prosthetic vein: 139, allograft vein: 14, other: 4), and the other 570 patients were included in the no vein graft group (end to end anastomosis: 544, lateral wedge 26) as the control.

### Meta-analysis of perioperative outcomes

Surgery duration data were available from 2 studies. Meta-analysis of these studies revealed that the vein graft group was associated with a significantly longer operation time (WMD = 87.04; 95% CI, 45.44–128.64; *P* < .001; *I*^2^= 0%). A meta-analysis of 3 studies indicated strikingly increased blood loss in patients with vein grafts (WMD = 509.47; 95% CI, 409.71–609.22; *P* < .001; *I*^2^ = 0%). Meta-analysis of 3 studies demonstrated significantly prolonged vascular clamp time for patients with vein grafts (WMD = 11.78; 95% CI, 8.93–14.64; *P* < .001; *I*^2^ = 0%). Subsequently, a meta-analysis of 6 studies with a total of 84 patients in the vein grafts groups and 231 patients in the no vein grafts group, indicated resected vein lengths to be notably longer in patients with vein grafts (WMD = 1.51; 95% CI, 1.24–1.78; *P* < .001; *I*^2^ = 64%). The sensitivity analyses excluded the study by Liao et al. [39], which resolved the heterogeneity (WMD = 1.99; 95% CI, 1.59–2.40; *P* < .001; *I*^2^ = 0%).

Data on perioperative morbidity were available from 3 studies, and meta-analysis of these studies indicated that perioperative morbidity was no different between the two groups (OR = 1.43; 95% CI, .75–2.73; *P* = .28; *I*^2^ = 15%). Meta-analysis of 1 study reveals that R0 resection rates was no different between the two groups (OR = 1.15; 95% CI, .40–3.29; *P* = .79). Meta-analysis of 8 studies demonstrated perioperative thrombosis not to be different between the vein graft group and the no vein graft group (OR = 1.38; 95% CI, 0.65–2.90; *P* = .40; *I*^2^ = 26%). A separate analysis was performed according to the different types of vein grafts, divided into the autologous vein subgroup and the prosthetic vein subgroup; no study reported data on the allograft vein group. Subgroup analysis demonstrated no difference in perioperative thrombosis between patents with autologous vein grafts and no vein grafts (OR = 0.78; 95% CI, 0.23–2.62; *P* = .69; *I*^2^ = 0%), as did the prosthetic vein subgroup (OR = 0.73; 95% CI, 0.23–2.31; *P* = .59; *I*^2^ = 19%).

Subsequent meta-analysis revealed that reoperation rate, pancreatic fistula, delayed gastric empty, hemorrhage, and biliary fistula were similar between the two groups (data not shown). Meta-analysis of 7 studies revealed no difference in the incidence of perioperative mortality in patients with and without grafts (OR = 1.43; 95% CI, 0.57–3.60; *P* = .44; *I*^2^ = 27%). The meta-analyses results are outlined in Table 2.

**Table 2:** Results of a meta-analysis comparing pancreaticoduodenectomy with and without grafts

Outcome of interest	No. of studies	vein graft group	no vein graft group	OR/WMD	95%CI	*P* value	*I^2^*	Meta-analysis model
**Perioperative outcomes**								
Operation time, min	2	48	156	87.04	45.44–128.64	< 0.0001	0%	Fixed
Blood loss, ml	3	63	183	509.47	409.71–609.22	< 0.00001	0%	Fixed
Clamp time,min	3	63	183	11.78	8.93–14.64	< 0.00001	0%	Fixed
lengths of resected vein,cm	6	84	231	1.91	1.59–2.40	< 0.00001	0%	Random
Overall morbidity	3	22/62	51/184	1.43	0.75–2.73	0.28	15%	Fixed
Perioperative thromboses	8	13/157	16/352	1.38	0.65–2.90	0.4	26%	Fixed
Perioperative mortality	7	6/122	11/278	1.43	0.57–3.60	0.44	27%	Fixed
**Long-term outcomes**								
Long-term thromboses	5	37/116	51/239	2.75	1.32–5.73	0.007	27%	Random
1-year overall surviavl	8	67/150	241/395	0.8	0.52–1.23	0.31	0%	Fixed
3-year overall surviavl	7	23/128	55/290	1.04	0.58–1.89	0.89	1%	Fixed
5-year overall surviavl	5	10/107	31/253	0.99	0.44–2.22	0.97	9%	Fixed

OR: odds ratio; WMD: weighted mean difference; CI: confidence interval.

### Meta-analysis of long-term function of interposition grafts

**Figure 2:**
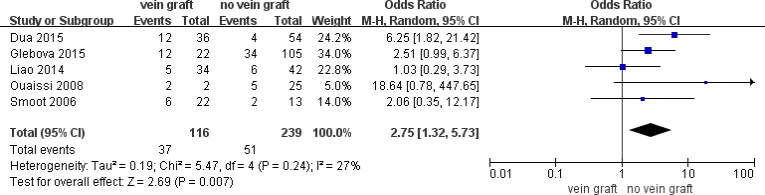
Meta-analysis of studies on long-term vein thrombose of patients undergoing pancretoduodenectomy with and without vein graft group by using random-effects model

**Figure 3:**
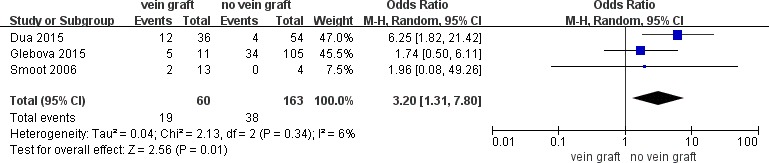
Meta-analysis of studies on long-term vein thrombose of patients undergoing pancretoduodenectomy with autologous vein graft versus no vein graft group by using random-effects model

To determine the long-term vein patency after reconstruction with vein grafts versus no vein grafts, comparative data was extracted and 5 studies were included in a meta-analysis of vein thrombosis (≥ 6 months) which exhibited a significant increase in the number of patients with vein grafts (OR = 2.15; 95% CI, 1.27–3.66; *P* = .005; *I*^2^ = 53%). The sensitivity analysis demonstrated that the heterogeneity was resolved by excluding the study by Leach et al. [39]. (OR = 2.75; 95% CI, 1.32–5.73; *P* = .007; *I*^2^ = 27%) (Figure 2). The autologous vein group subgroup analysis revealed no difference in vein thrombosis rate (≥ 6 months) compared to the no vein grafts group (OR= 1.95; 95% CI, 0.96–3.96; *P* = .06; *I*^2^ = 64%). Sensitivity analysis conducted by excluding the study by Leach et al [39] demonstrated a higher rate of vein thrombosis in patients with autologous vein grafts (OR = 3.20; 95% CI, 1.31–7.80; *P* = .01; *I*^2^ = 6%) (Figure 3). Meanwhile, the prosthetic vein group subgroup analysis indicated no difference in the vein thrombosis rate (≥ 6 months) between the prosthetic vein group and the no vein graft group (OR = 2.14; 95% CI, 0.98–4.69; *P* = .06; *I*^2^ = 0%) (Table 3).

**Table 3:** Subgroup analysis on perioperative and long-term thromboses during pancretoduodenectomy with and without concomitant vein graft

Varible	Subgroup	Perioperative outcomes	Long-term thromboses
The technique of PV-SMV reconstruction	autologous vein	OR = 0.78; 95% CI, 0.23–2.62; *P* = 0.69; *I*^2^ = 0%; *n* ***=*** 7	OR = 3.20; 95% CI, 1.31–7.80; *P* = 0.01; *I*^2^ = 6%; *n* ***=*** 3
	prosthetic vein	OR = 0.73; 95% CI, 0.23–2.31; *P* = 0.59; *I*^2^ = 19%; *n* ***=*** 6	OR = 2.14; 95% CI, 0.98–4.69; *P* = 0.06; *I*^2^ = 0%; *n* ***=*** 5

### Meta-analysis of pancreatic cancer survival

To evaluate the efficacy of vein graft technology during pancreatoduodenectomy for pancreatic cancer, the survival data of patients at 1, 3, and 5 years was extracted and analyzed. The meta-analysis indicated that there was no difference in overall survival in patients with and without grafts at 1 year (8 studies supported, OR = 0.8; 95% CI, 0.52–1.23; *P* = .31; *I*^2^ = 0%), 3 years (7 studies supported, OR = 1.14; 95% CI, 0.64–2.02; *P* = .65; *I*^2^ = 9%), or 5 years (5 studies supported, OR = 0.99; 95% CI, 0.44–2.22; *P* = .97; *I*^2^ = 9%) (Table 2).

## DISCUSSION

The present meta-analysis for the first time assesses the available data on the outcomes of patients who underwent PV-SMV reconstruction with vein grafts during PD. The findings revealed that although the duration of the operation, resected venous lengths, and clamping time were longer and blood loss was greater in patients with vein grafts undergoing PV-SMV reconstruction during PD than in patients without vein grafts, perioperative mortality, overall morbidity rates, and perioperative thrombosis were comparable between the groups. Moreover, there was no difference in the long-term survival of pancreatic cancer patients with and without grafts during PD. Therefore, it is clear that acceptable perioperative outcomes and long-term survival were achieved with both procedures.

Although primary end-to-end anastomosis or direct suturing are the preferred options for PV-SMV reconstruction without any interposition grafts, depending on the length, position, and extent of the resected segment of the PV-SMV, interposition grafts may be needed [5]. Our present study reported that primary end- to-end anastomosis was the most frequent procedure, performed in 570 patients (68.1%), followed by synthetic vein grafts in 131 patients (16.6%), autologous vein grafts in 110 patients (13.1%), then allograft vein grafts in 14 patients (1.7%), and other materials in 4 patients (0.5%). The type of autologous vein varied according to individual centers and was procured from separate operative sites. Numerous studies reported successful autologous vein grafts for PV-SMV reconstruction retrieved from various locations, such as the jugular vein [41, 42], the left renal vein [43–45], the external iliac vein [9, 46, 47], the femoral vein [48], and the great saphenous vein [33, 49]. For autologous vein grafts, it is important to select a graft with an optimal diameter and length to prevent graft occlusion [9, 46]. Several studies have focused on using prosthetic material, which mainly includes polytetrafluoroethylene (PTEE), as a graft for PV-SMV reconstruction [29, 30]. The PTEE grafts have a higher risk of infection compared with no grafts [50]. A PTEE graft can match various diameters and lengths and avoid additional autologous graft harvesting for PV-SMV reconstruction. There are very few studies reporting allograft vein graft for PV-SMV reconstruction. Roberto et al. reported that allograft vein graft for PV-SMV reconstruction did not require either ABO matching or immunosuppressive therapy [26]. Manju D. Chandrasegaram et al. reported that there is high heterogeneity in the use of anticoagulation policy, and the acceptable perioperative outcomes were achieved with an anticoagulation policy or no anticoagulation policy after venous resection [51].

Theoretically, the use of interposed grafts for PV-SMV reconstruction during PD is more effective than the external reinforcement ring, which helps maintain better perioperative long-term patency in the low-pressure portal system. The technique also can prevent over-tension or twisting of the reconstructed vein, which can lead to acute PV-SMV thrombus. The present study demonstrated no difference in perioperative thrombosis in patients with vein grafts undergoing PV-SMV reconstruction during PD compared to patients without vein grafts, and the subgroup analysis also showed no difference. However, the lower long-term (≥ 6 months) vein patency rate in the vein graft group indicates that interposition grafts may be more likely to lose function and result in vein occlusion. The risk of thrombosis can be due to exposure of graft material at the endothelial surface, and vein grafts expose blood flow to grafts at two suture lines, while a primary anastomosis only requires one suture line. Furthermore, subgroup analysis indicated that using a prosthetic vein seems more effective than using an autologous vein in PV-SMV reconstruction during PD to maintain long-term PV-SMV patency. This result could be attributed to that a PTEE graft can match various diameters and lengths for PV-SMV reconstruction, and an autologous vein may be related to high fibrinogen levels and low protein C levels [51].

The present study has several limitations. All meta-analysis data came from non-randomized controlled trials, and the overall level of clinical evidence is low. Randomized assessment of interposition grafts for PV-SMV reconstruction during PD is difficult because of ethical reasons. Furthermore, we failed to retrieve some important data from the original authors, including oncological and long-term morbidity; therefore, some selection bias may still exist.

In conclusion, the present study provides evidence that using interposition grafts for PV-SMV reconstruction can achieve perioperative outcomes and long-term survival that were comparable to those with no vein grafts during PD with VR and can be performed safely and effectively, which considering a more aggressive approach to increased resection rates. However, the technique of using grafts for PV-SMV reconstruction affects the long-term vein patency rate, indicating that interposition grafts may be more likely to lose function and result in vein occlusion, and prosthetic veins are more effective than autologous veins in PV-SMV reconstruction during PD to maintain long-term PV-SMV patency.

## MATERIALS AND METHODS

### Search strategy

A computerized search of the PubMed, Embase, Web of Science, ClinicalTrial.gov and Cochrane Library databases was made of all articles published between January 1963 and July 2016. The search was restricted to studies on humans published in the English language. The following terms search terms were used: pancreaticoduodenectomy,” “pancreatoduodenectomy,” “duodenopancreatectomy,” “pancreatectomy,” “pancreatic resection,” “vein resection,” “vascular resection,” “vein reconstruction,” “vascular reconstruction,” “vein grafts,” “vascular grafts,” and “grafts.” The reference lists of all relevant articles obtained were screened manually to identify potentially eligible studies. If there was any doubt about the suitability of the studies after reading the titles and abstracts, the full articles were obtained for detailed evaluation, and all eligible studies were included.

### Data extraction

Two authors (Wei Song and Qif Yang) independently extracted the following categories from each full study: first author, year of publication, study period, study design, number of patients treated with each procedure, operative data (including duration of surgery, time of vein clamping, estimated blood loss, length of vein resected, reconstruction techniques, type of vein grafts), postoperative morbidity and mortality, and histopathology. To assess the value of the reconstruction techniques, we extracted pancreatic cancer data, including the median survival and survival rates at 1, 3, and 5 years after surgery. All relevant comparative data were reviewed for data extraction. In addition, we also wrote emails to the original authors to request some missing key data.

### Inclusion and exclusion criteria

For each included study in the meta-analysis, a study had to satisfy the following criteria: compare the results of pancreaticoduodenectomy with vein grafts after vein resection (autologous vein, prosthetic vein, allograft vein) versus without vein grafts (primary end-to-end anastomosis, lateral wedge) in patients undergoing vascular resection surgery; report at least one of the outcomes of interest listed below; and when two or more studies were published from the same institution, the higher quality study was included in the analysis. Abstracts, letters, editorials, expert opinions, case reports, reviews without original data, and studies without control groups were excluded.

### Quality assessment

We used the Newcastle-Ottawa Quality Assessment Scale (NOS) bias risk tool to assess the methodological quality of the included studies [40]. Each section was judged according to the appropriate definition, and those with a score ≥ 7 were considered high quality and included in our study.

### Statistical analysis

Meta-analysis of the extracted data was performed using Cochrane Review Manager 5.3. (http://ims.cochrane.org/revman). Odds ratio (OR) was chosen to calculate dichotomous data with 95% confidence interval (CI), and weighted mean difference (WMD) for continuous data with 95% CI. A fixed-effects model or random-effects model was used, depending on the absence or presence of heterogeneity. The statistical heterogeneity was evaluated by the *Q* test (x^2^) and statistic with significance set at *P* < .05 and < 50%. Sensitivity analyses were performed to determine the effect of outliers by excluding some unique studies. Publication bias was assessed using visual examination with a funnel plot.

## Abbreviations

NOS: Newcastle-Ottawa Quality Assessment Scale
OR: odds ratio
PD: pancreatoduodenectomy0020
PTEE: polytetrafluoroethylene
PV-SMV: portal-superior mesenteric vein
VR: venous resection
WMD: weighted mean difference
